# Ocular myasis and associated mucopurulent conjuctivitis acquired occupationally: A case study

**DOI:** 10.4103/0019-5278.40811

**Published:** 2008-04

**Authors:** K. Jayaprakash, A. Karthikeyan

**Affiliations:** Department of Environmental Science, JJ College of Arts and Science, (Bharathidasan University, Tiruchirappalli), Pudukkottai - 622 404, Tamil Nadu, India; 1 Department of Microbiology, JJ College of Arts and Science, (Bharathidasan University, Tiruchirappalli), Pudukkottai - 622 404, Tamil Nadu, India

**Keywords:** Mucopurulent conjuctivitis, occupational ocular myasis, oestrus ovis

## Abstract

Ocular myasis and associated mucopurulent conjunctivitis in human eyes is a rare phenomenon. However, if the sheep bot fly abounds and poor hygienic environment prevails, the *Oestrous ovis* deposits its larvae in the conjunctival eye sac of human. The present paper reports a case study of ocular myasis among sheep farm workers caused by *Oestrous ovis*. The ocular myasis and the associated mucopurulent conjunctivitis are occupationally acquired in these cases. This study also suggests the treatment of patients and the recovery of the larvae.

## INRODUCTION

In India, agriculture is a major profession. In the agricultural sector, the laborers engaged in field activities may be exposed to several organisms causing zoonotic diseases. The breeding and rearing of sheep or goats is also one such agricultural based activity in India. An unorganized labor force is deputed by the farmers for taking care of animals in sheep farm. These laborers may have the chances of zoonosis due to insects in the vicinity of sheep farm. There has been prevalence of ocular myasis associated with mucopurulent conjunctivitis among these workers. Ocular myasis is due to the infestation of the eye with maggots or bots of certain flies.[1] The infection is either specific or facultative. Although myasis in man is generally rare, members of *Cyclorhapid* and *Oestridae* may produce myasis. The *Oestrous ovis*, the common sheep bot fly breeds in the nasal cavity and sinuses of sheep. The fly enters the nostrils and deposits its larvae. The larva crawls and reaches the brain cavities, they mature and fall on the ground and become adult. In the sheep farm the standard of hygiene is always very low and there are no techniques adopted to stop the activities of these insects.[2,3] We have been observing frequent incidences of occupational ocular myasis of such kind in workers of sheep farm.

The present paper describes the case study on the pathology, treatments and associated microbes for the development of mucopurulent conjunctivitis acquired occupationally in the sheep farm workers.

## MATERIALS AND METHODS

The sheep farm worker infected with first stage larvae of *Oestrous ovis* in eye and eye discharge due to mucopurulent conjunctivitis were the cases examined. The infected eye was observed by ophthalmoscope. The collected discharge namely serous, mucopurulent was analyzed for the presence of pathogens by culture techniques. The bacterial strains isolated and characterized by staining techniques. Antibiotic sensitivity pattern of the organism was studied by Kirby-Bauer method.

The antibiotics such as garamycin, neomycin, erythromycin, tetracycline, ofloxacin, norfloxacin and sulfacetamide were tested. The recovery and the removal of insect larvae *in question* was done by immobilizing with the topical application of 1% xylocaine and grasped with the help of forceps. The diseased eyes were treated with suitable anti-inflammatory drugs and antibiotics determined out of the culture studies.

## RESULTS

**Figure 1:**
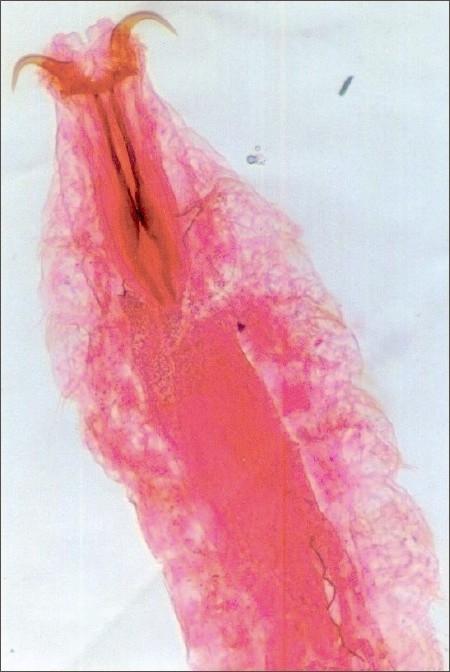
Head of the larvae enlarged showing barks and bristles of the body

**Figure 2:**
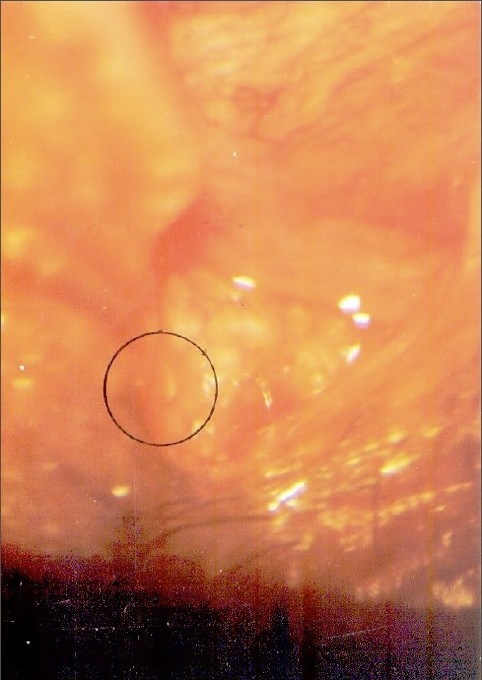
Mucopurulent conjunctivitis in the eye with larvae *in situ*

**Figure 3:**
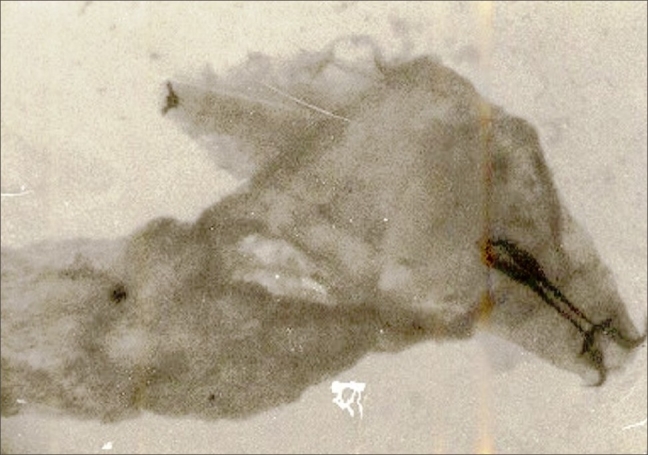
Mucopurulent serous with larvae

The observation on the infected eyes of cases (workers of sheep farm) through the slit lamp revealed the presence of larvae of *Oestrous ovis*. These larvae caused tissue damage over the eye ball and conjunctival sac. The movement of the larvae was affected by the body bristles and barbs [Figure 1]. There was an evidence for the tissue erosion on the conjunctival epithelium and on the surface of the eye ball. The larva freely crawls on the eye ball and thrived in the eye fluid. There was also an indication of severe mucopurulent and serosa discharge from the infected eyes [Figures 2 and 3]. Further the observation also disclosed the symptoms of redness of the eye, watering, photophobia and swellings. The culture study performed with the discharge of the infected eye indicated the presence of pathogens such as *Staphylococcus aureus, Staphylococcus epidermidis, Pseudomonas* and *Moraxella* sp. The important observation was the presence of *Chlamydiae trachomatis* in certain cases.

These results suggested that the infection with *Oestrous ovis* larvae is associated with mucopurulent conjunctivitis causing eye pathogens. The degree of mucupurulent conjunctivitis depends on the nature of tissue damage caused by the larva. The results of antibiotic disc sensitivity test exhibited the sensitivity to various antibiotics [Table 1]. Among the seven antibiotics ofloxacin was found strong inhibition on the pathogens.

**Table 1:** Results of antibiotic disc sensitivity test on the cultures of mucopurulent discharge

Name of the antibiotics	Eye Pathogens						
	
	*Staphylococcus* sp.	*Staphylococcus epidermidis*	*P*seudomonas sp.	*Moraxella* sp.	*Streptococcus* sp.	*Chalmydia trachomatis*	*Oestrous ovis* larvae
Garamycin	R	R	S	MS	R	R	R
Neomycin	MS	MS	MS	MS	MS	MS	R
Erythromycin	MS	MS	MS	MS	MS	MS	R
Tetracycline	MS	MS	MS	MS	MS	MS	R
Ofloxacin	S	S	S	S	S	S	S
Norfloxacin	S	S	S	S	S	S	S
Sulfacetacmide	MS	MS	MS	MS	MS	MS	S

R - Resistant; MS - Moderately susceptible; S - Susceptible

## DISCUSSION

The ocular myasis due to the infection of first stage larvae of sheep bot fly associated with mucopurulent conjunctivitis in human eyes is a rare occurrence. However if the sheep bot fly *Oestrous ovis* is abound, there may be chances of deposition of larvae in the conjunctival sac of the human eyes. The incidences of ocular myasis associated with mucopurulent conjunctivitis described in the present cases may be occupationally contracted. The erosion of epithelial tissues on the eye ball as well as conjunctival cavity may be prone for the infection of microbes.[4] This is evidenced from the analysis of cases reported in this present study.

The treatment suggested out of the present study for the recovery and eradication of the conjunctivitis may be curative. The occupationally acquired mucopurulent conjunctivitis among the sheep farm workers may be the first of this kind. It has been known that the activities of the said fly are high in sheep farm. The fly repellents (or) fumigation of insecticides are not very effective. It has been suggested that injection of ivermectin, doramectin or moxidectin into the nostril of the sheep may be effective against the larvae and the control of adult flies.[5]

The significant observation in this context is the presence of *Chlamydiae trachomatis* which is recently known for causing blindness.[6] It is desirable to investigate further to ascertain the pathology of this occupationally contracted mucopurulent conjunctivitis in these workers on environmental basis.
